# Zebrafish IGF Genes: Gene Duplication, Conservation and Divergence, and Novel Roles in Midline and Notochord Development

**DOI:** 10.1371/journal.pone.0007026

**Published:** 2009-09-17

**Authors:** Shuming Zou, Hiroyasu Kamei, Zubin Modi, Cunming Duan

**Affiliations:** 1 Department of Molecular, Cellular, and Developmental Biology, University of Michigan, Ann Arbor, Michigan, United States of America; 2 Key Laboratory of Aquatic Genetic Resources, Shanghai Ocean University, Shanghai, China; 3 Laboratory of Molecular Medicine, School of Medicine and Pharmacy, Ocean University of China, Qingdao, China; Harvard University, United States of America

## Abstract

Insulin-like growth factors (IGFs) are key regulators of development, growth, and longevity. In most vertebrate species including humans, there is one IGF-1 gene and one IGF-2 gene. Here we report the identification and functional characterization of 4 distinct IGF genes (termed as *igf-1a*, *-1b*, *-2a*, and *-2b*) in zebrafish. These genes encode 4 structurally distinct and functional IGF peptides. IGF-1a and IGF-2a mRNAs were detected in multiple tissues in adult fish. IGF-1b mRNA was detected only in the gonad and IGF-2b mRNA only in the liver. Functional analysis showed that all 4 IGFs caused similar developmental defects but with different potencies. Many of these embryos had fully or partially duplicated notochords, suggesting that an excess of IGF signaling causes defects in the midline formation and an expansion of the notochord. IGF-2a, the most potent IGF, was analyzed in depth. IGF-2a expression caused defects in the midline formation and expansion of the notochord but it did not alter the anterior neural patterning. These results not only provide new insights into the functional conservation and divergence of the multiple *igf* genes but also reveal a novel role of IGF signaling in midline formation and notochord development in a vertebrate model.

## Introduction

Insulin-like growth factors (IGFs), including IGF-1 and IGF-2, are evolutionarily conserved peptides that act through a conserved signaling pathway to regulate growth, development, metabolism, and longevity in a wide variety of animals [1]. IGF-1 and -2 are structurally related to insulin and relaxin in vertebrates, and insulin-like peptides (ILP) in invertebrates [2], [3], [4], [5]. IGFs and insulin share 50% sequence identity in amino acid sequence in their B and A domains. Unlike insulin, however, the C-domains of IGFs are not removed by proteolytic processing. In addition, IGF-1 and IGF-2 contain an E- domain in their carboxy-terminus. The E-domain is removed as a post-translational modification to produce mature IGFs [6]. Activation of the IGF signaling pathway occurs when IGF ligands bind their cognate receptor tyrosine kinases. This leads to activation of a number of downstream signaling cascades, including mitogen-activated protein kinase (MAPK) and phosphatidylinositol 3-kinase (PI3K)-Akt pathways [7], [8], [9].

In mammals and birds, there is a single IGF-1 gene and a single IGF-2 gene, in addition to a single insulin gene. Recent studies have indicated the zebrafish genome contains more than two IGF genes. Chen et al. (2001) reported the presence of a gene encoding an IGF-like peptide [10]. Maures at al. (2002) cloned cDNAs encoding the complete coding sequences for the same IGF-1-like peptide and an IGF-2 peptide [5]. A more recent study by Sang et al. (2008) reported the presence of two distinct IGF-2 genes (*igf-2a* and *-2b*) and that these genes exhibited distinct expression patterns [11]. They further showed that knockdown of *igf-2a* and *-2b* by antisense morpholinos indicated that they each play a distinct role in early development [12]. Wang et al. (13) reported the cloning of another IGF peptide that they termed as IGF-3, from tilapia and zebrafish [13].

Despite these new findings, there has been no report on the molecular characterization of any of these zebrafish IGF genes or their full-length transcripts. For instance, little is known about the alternative splicing for any of the previously identified zebrafish IGF genes, although alternative splicing has been shown to be an important way of generating multiple forms of IGF transcripts and prepropeptides in mammals and humans. Moreover, zebrafish, like many teleost fish, are believed to have experienced an additional genome wide duplication event [14], [15]. As a result, they often have two co-orthologs in contrast to a single copy gene in humans and other mammals. Indeed, there are two distinct insulin genes, two IGF-1R genes, and two insulin receptor genes in zebrafish [5], [16], [17], [18]. To date, there is no report on the possible presence of the fourth IGF gene in zebrafish. In this study, we have cloned and identified 4 distinct genes encoding 4 IGF peptides (IGF-1a, -1b, -2a, and -2b) from zebrafish. The structures of these 4 zebrafish IGF genes and their transcripts have been determined. Our molecular and functional analyses suggest that these IGF genes have undergone subfunctionalization partitioning at the levels of gene expression, protein structure, and biological activities. Furthermore, taking advantage of the amenability of the zebrafish model, we unraveled a previously unrecognized role of IGF in regulating midline and notochord development during embryogenesis *in vivo*.

## Results

### Identification and characterization of 4 genes encoding distinct IGF peptides in zebrafish

**Figure 1:**
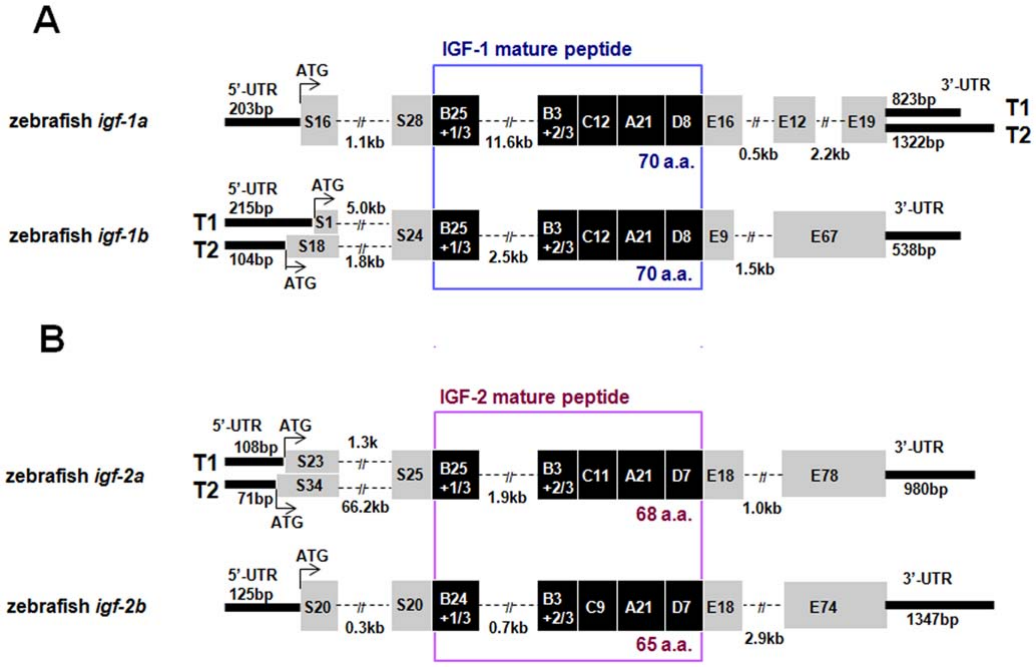
Molecular characterization of four zebrafish *igf* genes and their multiple transcripts. (A) Structure of the zebrafish *igf-1a* and *-1b* genes. Signal peptide and E domain regions are shown in grey boxes and the B–C–A–D domains of the mature peptide are shown in dark boxes with the number of amino acids indicated. 3′ and 5′-UTRs are shown in bold lines with the number of base pairs shown (bp). Introns sequences are shown in dashed lines and are indicated in kb. Two alternative splicing transcripts (T1 and T2) are found in the 3′-UTR of *igf-1a*. Two alternative splicing transcripts are also found in the 5′-UTR and signal peptide region of *igf-1b*. (B) Structure of the zebrafish *igf-2a* and *igf-2b*. Two alternative splicing transcripts are found in the 5′-UTR and signal peptide region of *igf-2a*.

By searching available zebrafish databases and performing 5′ and 3′RACE experiments, we have identified 4 genes and a number of full-length cDNAs encoding 4 structurally distinct IGF-like peptides (IGF-1a, -1b, -2a, and -2b). The structure of these zebrafish *igf* genes and their transcripts are shown in Fig. 1. Zebrafish *igf-1a* has 5 exons and 4 introns and it spans approximately 17 kb. Two distinct *igf-1a* mRNA transcripts (T1, 1509 bp and T2, 2008 bp) were found (Fig. 1A). These *igf1a* transcripts have an identical open reading frame of 483 bp encoding a polypeptide of 161 amino acids (a.a.). This peptide can be divided into a 44 a.a. putative signal peptide, a 29 a.a. B domain, a 12 a.a. C domain, a 21 a.a. A domain, a 8 a.a. D domain, and a 47 a.a. E domain. T1 and T2, likely resulted from alternative splicing, have distinct 3′ UTR of 823 and 1322 bp, respectively (Fig. 1A). Zebrafish *igf-1b* (9.1 kb and 5.9 kb) has 4 exons and it has 2 different IGF-1b transcripts (T1, 1269 bp and T2, 1209 bp). The full-length *igf-1b* T1 includes an open reading frame of 513 bp, which encodes a polypeptide of 171 a.a. (containing a 25 a.a. signal peptide, a 76 a.a. E domain, and the B–C–A–D domain). The *igf-1b* T2 differs from T1 only in the 5′ UTR and a portion of the signal peptide (Fig. 1A). Zebrafish *igf-2a* has 4 exons and spans 5.9∼70.8 kb DNA (Fig. 1B). Two *igf-2a* transcripts (1727 and 1723 bp) were identified. *igf-2a* T1 and T2, likely resulted from alternative splicing sites in the exon 1 and exon 2, differ in the 5′ UTR and part of the signal peptide region. The open reading frame for *igf-2a* T1 and T2 are 636 bp and 669 bp, respectively. They encode polypeptides of 212 and 223 a.a., which containing a 48/59 a.a. signal peptide, a 29 a.a. B domain, an 11 a.a. C domain, a 21 a.a. A domain, a 7 a.a. D domain, and a 96 a.a. E domain (Fig. 1B). Zebrafish *igf-2b* has 4 exons and spans about 5.9 kb. Only one transcript was found for this gene. The full-length cDNA is 2066 bp. It contains an open reading frame of 591 bp encoding a polypeptide of 197 a.a., including a 40 a.a. signal peptide, a 28 a.a. B domain, a 9 a.a. C domain, a 21 a.a. A domain, a 7 a.a. D domain, and a 92 a.a. E domain (Fig. 1B).

**Figure 2:**
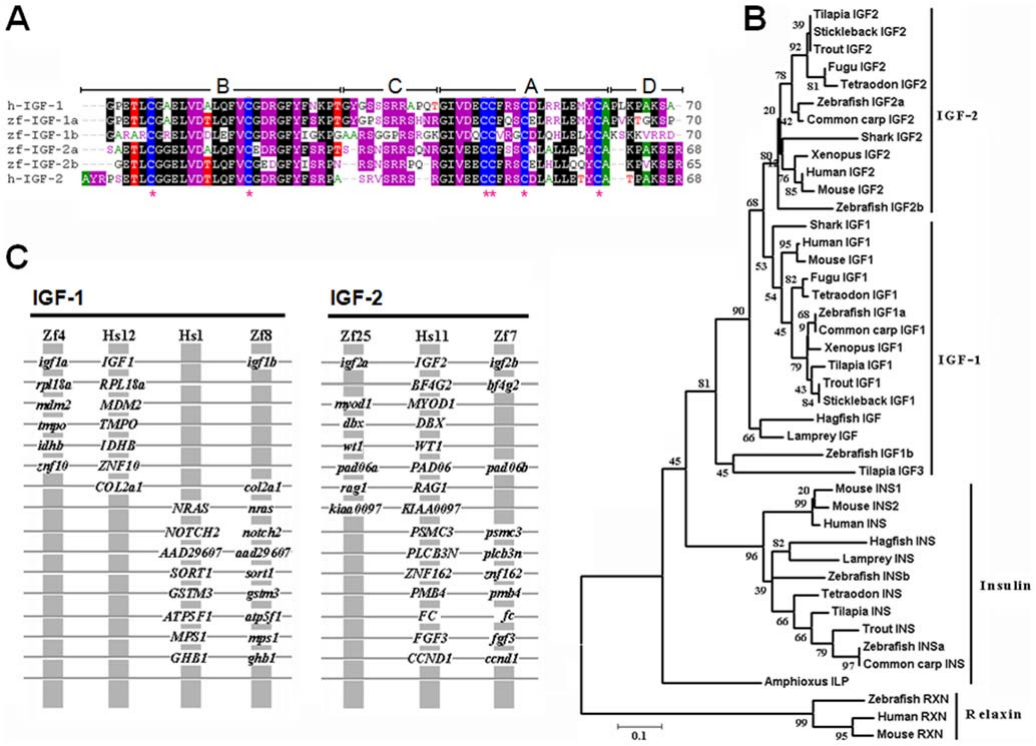
Structural and phylogenic analysis of four zebrafish IGFs. (A) Sequence alignment of mature zebrafish (zf) IGF-1a, IGF-1b, IGF-2a, IGF-2b and mature human (h) IGF-1 and IGF-2. The B–C–A–D domains are labeled. Star indicates conserved cysteine. (B) Phylogenetic analysis of the vertebrate insulin/IGF family members. Amino acid sequences in the B and A domains were analyzed by the neighbor-joining method using the MEGA3 program [64]. Gaps were removed from the alignment. Bootstrap values derived from 1,000 runs are shown. Relaxins are used as an outgroup. Accession numbers for the sequences used are: human (*IGF-1*, NM_000618; *IGF-2*, NM_000612; *insulin*, NM_000207; *relaxin*, NM_080864), mouse (*IGF-1*, NM_010512; *IGF-2*, NM_010514, *insulin* 1, NM_008386; *insulin 2*, NM_008387; *relaxin*, NM_173184), *Xenopus* (*IGF-1*, M29857; *IGF-2*, BC070545), zebrafish (*insulin a*, NM_131056; *insulin b*, NM_001039064), common carp (*IGF-1*, EF536889; *IGF-2*, AF402958; *insulin*, X00989), trout (*IGF-1*, EF432852; *IGF-2*, EF432854; *insulin*, M21170), tilapia (*IGF-1*, AF033796; *IGF-2*, AF033801; *IGF-3*, EU272147; *insulin*, AF038123 ), shark (*IGF-1*, Z50081; *IGF-2*, Z50082), hagfish (*IGF*, M57735; *insulin*, V00649 ), sea lamprey (*IGF*, AB081462), Amphioxus insulin-like peptide mRNA (*ILP*, M55302). Sequence for *Tetraodon*, fugu and stickleback IGF-1, IGF2 and insulin were obtained by searching the *Tetraodon* genome (http://www.genoscope.cns.fr/externe/English/Projets/Projet_C/C.html), fugu genome (http://fugu.hgmp.mrc.ac.uk/), and stickleback genome (http://www.ensembl.org/ Gasterosteus_aculeatus/). Sequence of sea lamprey insulin was obtained by searching sea lamprey genome (http://pre.ensembl.org/Petromyzon_marinus/). (C) Conserved synteny between human (Hs) and zebrafish (Zf) IGF loci. Vertical gray lines indicate a group of genes on the same chromosome, with order ignored to facilitate the comparison of orthologs and paralogs. Horizontal gray lines connect presumed orthologs within chromosome groups as well as paralogs between chromosome groups.

The 4 zebrafish IGF genes and their various mRNAs encode 4 distinct mature IGF peptides, IGF-1a (70 a.a.), IGF-1b (70 a.a.), IGF-2a (68 a.a.), and IGF-2b (65 a.a.). These peptides are highly similar to human IGFs in their primary structure (Fig. 2A). Like human IGFs, all 4 zebrafish IGF peptides consisted of 4 domains (B–C–A–D). They all possess 6 conserved cysteine residues: 2 in the B domain and 4 in the A domain region. Phylogenetic analyses grouped all four zebrafish IGF peptides into the IGF clade, indicating that they are IGF like molecules rather than insulin or relaxin related peptides (Fig. 2B). Zebrafish IGF-2a and IGF-2b are clearly more closely related to human IGF-2 because they share equally high sequence identities with that of human IGF-2 (76% and 75%, respectively) and a 70% sequence identity with each other. Phylogenetic analysis also grouped them into the IGF-2 subgroup. Zebrafish IGF-1a and IGF-1b are more divergent because they are only 50% identical with each other. While zebrafish IGF-1a shares 81% sequence identity to that of human IGF-1, the sequence identity between zebrafish IGF-1b and human IGF-1 is much lower (52%). Phylogenetic analysis also placed IGF-1b in a separate group - together with the recently reported tilapia IGF-3 [13] (Fig. 2B).

The duplicated zebrafish *igf-2* genes share strong synteny with the human *IGF-2*. As shown in Fig. 2C, zebrafish *igf-2a* and *igf-2b* are located on LG 25 and 7, respectively. There are a number of genes on LG25 and 7 whose orthologs are located on human chromosome 11, where the human *IGF-2* is found. Zebrafish *igf-1a* is located on LG4. There are five other zebrafish genes (*rpl18a*, *mdm2*, *impo*, *idhb*, and *znf10*) in this region whose orthologs are located on human chromosome 12, where the human *IGF-1* resides. The zebrafish *igf-1b* gene, on the other hand, is located on LG8. Although we were able to find 1 gene (*COL2a1*) in this region whose orthologous gene is located on human chromosome 12, 8 other zebrafish genes (*nras*, *notch2*, *aad29607*, *sort1*, *gstm3*, *atp5f1*, *mps1*, and *ghb1*) on LG8 have orthologs located on human chromosome 1 (Fig. 2C).

### All 4 zebrafish *igf* genes are functional and they exhibit divergent expression patterns

**Figure 3:**
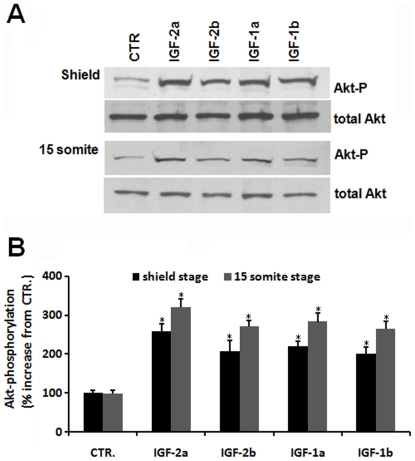
All four zebrafish *igf* genes are functional. (A) Microinjection of each of the 4 zebrafish IGF mRNA into zebrafish embryo resulted in elevated Akt phospholylation. GFP, IGF-1a, -1b, -2a, and -2b mRNA was injected into zebrafish embryos at 1-2 cell stage. The injected embryos were raised to shield and 15 somite stages. Lysates were prepared and subjected to SDS-PAGE followed by immunoblot analysis using antibodies against total and phosphorylated Akt (Akt-P). (B) Densitometric analysis result of (A). The phosphorylated Akt/total Akt ratio was calculated and expressed as % of the control (GFP mRNA injected) group. Grey bars represent the shield stage groups, and dark bars represent the 15 somite stage groups. Values are means±SE (n = 3). *p<0.01 compared with the control group.

To determine whether all 4 zebrafish *igf* genes are functional, capped mRNA encoding each of the zebrafish IGF genes was generated and delivered to zebrafish eggs by microinjection. Their effects on Akt phosphorylation were analyzed by Western blot. Akt is a major downstream effector of the IGF1R in mammals and in zebrafish [19]. Western blot analysis indicated a marked increase in the levels of phosphorylated Akt in all IGF mRNA-injected groups compared to the GFP mRNA-injected controls at both shield and 15 somite stages (Fig. 3A). When quantified and expressed as the ratio of phospho-Akt/total Akt, all of the 4 IGF mRNAs caused significant increases (Fig. 3B), indicating that they are all capable of interacting with IGF-1R and activating the intracellular signaling cascade.

**Figure 4:**
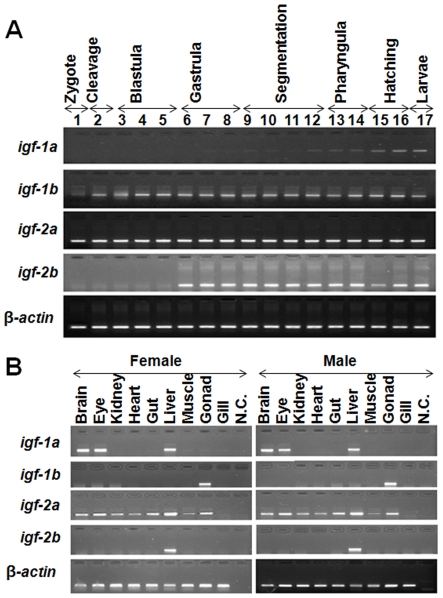
Temporal and spatial expression patterns of the duplicated zebrafish IGF genes. (A) RT-PCR analysis of IGF mRNAs in zebrafish embryos at the indicated stages. Numbers are the different embryo developmental stages (1, 1–2 cell; 2, 32-cell; 3, 512-cell; 4, oblong; 5, 30%-epiboly; 6, 50%-epiboly; 7, 90%-epiboly; 8, tailbud; 9, 2-somite; 10, 12-somite; 11, 18-somite; 12, 24–26 somite; 13, Prim-6; 14, Prim-22; 15, 48hpf; 16, 72hpf; 17, 96hpf). (B) RT-PCR analysis of IGF mRNAs in female and male adult zebrafish tissues.

Next, the temporal and spatial expression patterns of the 4 zebrafish *igf* genes were examined. As shown in Fig. 4A, IGF-1a mRNA was not detectable by RT-PCR in early embryogenesis. IGF-1a mRNA became detectable at the gastrulation stage (around 90%-epiboly). Its level gradually increased after 48 hpf. Likewise, IGF-2b mRNA was detected at 50%-epiboly and was maintained at relatively high levels thereafter (Fig. 4A). In comparison, both IGF-1b and IGF-2a mRNAs were easily detected in all stages of embryogenesis, ranging from the 1-cell stage to 4 dpf larvae (Fig. 4A). These data suggest that IGF-1b and -2a transcripts are maternally deposited, while IGF-1a and -2b mRNAs are the products of the zygotic genes activated after the mid-blastula transition. In the adult stage, IGF-1a mRNA was easily detectable in the liver, brain, and eyes in both male and female fish, while IGF-1b mRNA was only detected in the gonad (Fig. 4B). IGF-2a mRNA was easily detected in all the tissues examined, although its levels in the muscle and gills were very low (Fig. 4B). In contrast, IGF-2b mRNA was exclusively expressed in the liver (Fig. 4B). There was no apparent gender difference.

In agreement with the RT-PCR data, whole mount *in situ* RNA hybridization analysis showed that IGF-1a and IGF-2b mRNA was very low at 10 hpf, became detectable at 24 hpf, and highly expressed at 48 hpf, most abundantly in the anterior part of a embryo (data not shown). In comparison, IGF-1b and IGF-2a mRNA was detected in all stages in a wide range of tissues (data not shown).

### Forced expression of zebrafish IGFs causes similar defects in midline formation and notochord development but with different potencies

**Figure 5:**
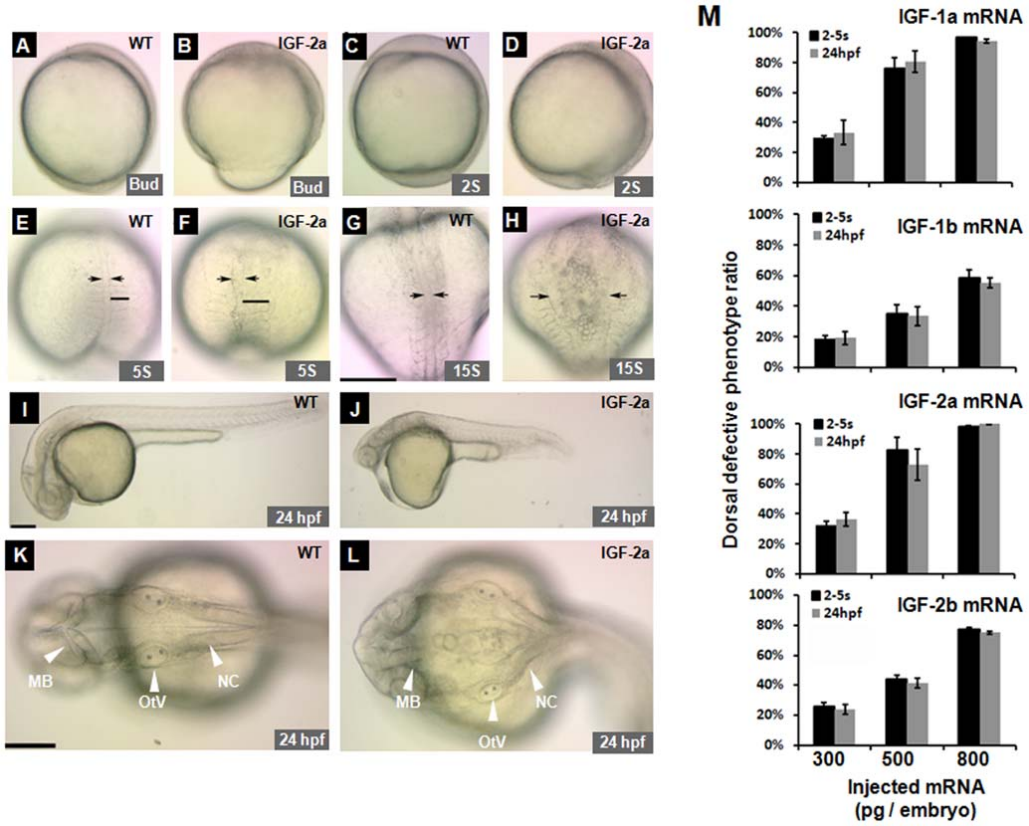
Effect of forced IGF expression in zebrafish embryos. (A, B) Lateral view of a GFP mRNA injected control embryo (A) and an IGF-2a mRNA injected embryo (B) at the bud stage. Note the delayed mesoderm involution associated with an open blastopore (>75%, n = 328) in the IGF-2a mRNA injected embryos (B). (C, D) Lateral views of a control embryo (C) or an IGF-2a mRNA injected embryo (D) at 2-somite stage. Note the shortened A-P axis and more posterior tissues in (D). (E–H) Dorsal view of a control (E, G) or an IGF-2a mRNA injected embryo (F, H) at the 5-somite (E and F) and 15-somite stages (G and H). All embryos are dorsal views with head up. Arrows indicate the width of the notochord. Black bars in panels E and F show the width of the somite. Scale bar  = 200 µm. (I–L) Morphology of a GFP mRNA injected control embryo (I and K) and an IGF-2a mRNA injected embryo (J and L) at 24 hpf. I and J are lateral views with head to the left and K and L are dorsal views with head to the left. MB, mid brain; OtV, otic vesicle; NC, notochord. Scale bar  = 200 µm. (M) Dose-dependent effects of various IGFs in zebrafish embryos. The results are means of 3–4 independent experiments.

The phenotypes of the IGF-2a mRNA (500 pg)-injected group are shown in Fig. 5A–L. Injection of IGF-2a mRNA (500 pg) resulted in delayed mesoderm involution as evident from an open blastopore at the bud stage (Fig. 5B). At the 2-somite stage, there was a notable reduction in the posterior tissues (Fig. 5D). At the 5-somite stage, the IGF-2a mRNA-injected embryos showed poorly formed notochords and widened distance between the two columns of somites (Fig. 5F). This defect became more evident at the 15-somite stage (Fig. 5H). Compared to the control (Fig. 5K), the IGF-2a mRNA-injected embryos showed marked expansion of dorsal neural tissues and axial domain (Fig. 5L). Additionally, these embryos showed a severe shortening body length (Fig. 5J).

To determine whether these IGFs have similar biological activities, each IGF mRNA was injected at the doses of 300, 500, and 800 pg/embryo and percentages of embryos exhibiting the above phenotype were determined at the 2–5 somite stage and at 24 hpf. Zebrafish IGF-1a, -1b, -2a and -2b all caused similar defects in dose-dependent manners (Fig. 5M), but there were considerable differences in their effectiveness. Injection of IGF-2a at 300 pg/embryo resulted in a score value of 33%∼35%; a score value of 75%∼80% at 500 pg; and over 90% at 800 pg/embryo at the 2–5 somite and 24hpf stages. Similar results were obtained with the injection of IGF-1a mRNA and to a lesser degree with IGF-2b mRNA (Fig. 5M). In comparison, IGF-1b appeared to have a weaker effect. It only had a score of 20% at the dose of 300 pg/embryo, a score of <40% at 500 pg, and <60% at 800 pg/embryo (Fig. 5M).

### Forced expression of IGF-2a disrupts midline and notochord patterning

**Figure 6:**
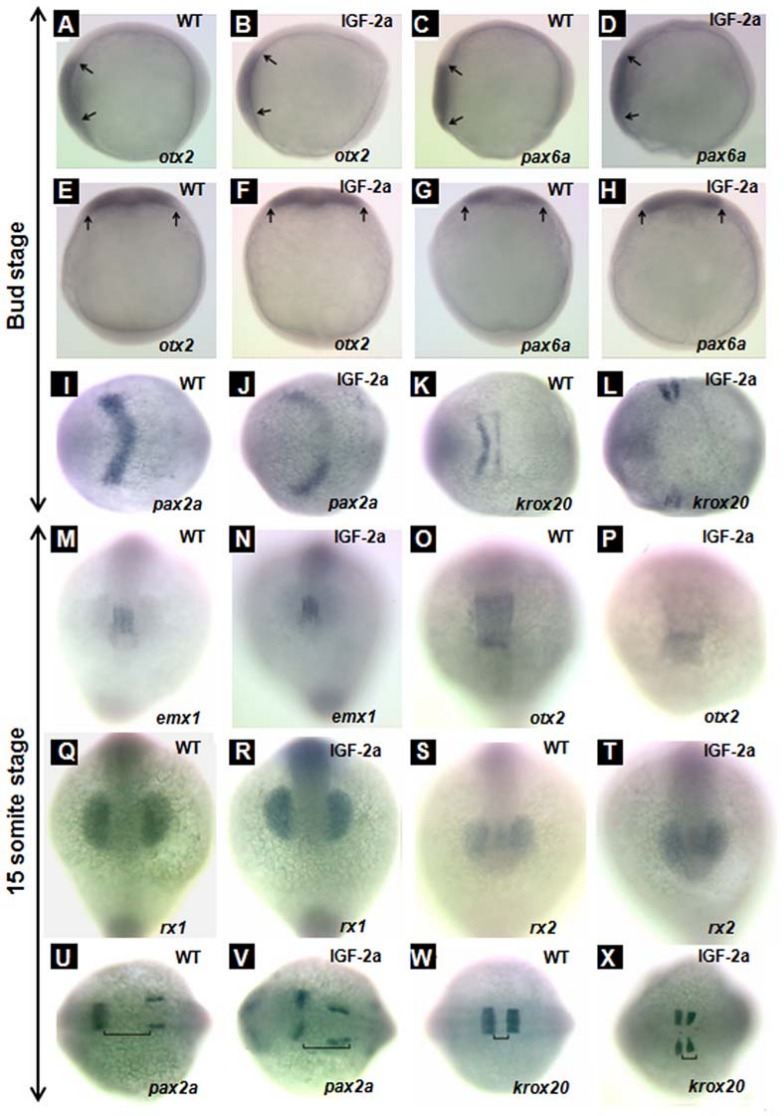
Effect of IGF-2a expression. *otx2* expression in the anterior neural plate at the bud stage in a control embryo (panels A and E) and an IGF-2a mRNA injected embryo (panels B and F). *pax6a* expression in the anterior neural plate at the bud stage in a control embryo (panels C and G) and an IGF-2a mRNA injected embryo (panels D and H). *pax2a* expression in the midbrain hindbrain boundary at the bud stage in a control embryo (panel I) and an IGF-2a mRNA injected embryo (panel J). *krox20* expression in the rhombomere 3 and 5 at the bud stage in a control embryo (panel K) and an IGF-2a mRNA injected embryo (panel L). *emx1* expression in the forebrain at the 15-somite stage in a control embryo (panel M) and an IGF-2a mRNA injected embryo (panel N). *otx2* expression in the midbrain at the 15-somite stage in a control embryo (panel O) and an IGF-2a mRNA injected embryo (panel P). *rx1* expression in the optic vesicle at the 15-somite stage in a control embryo (panel Q) and an IGF-2a mRNA injected embryo (panel R). *rx2* expression in the optic vesicle at the 15-somite stage in a control embryo (panel S) and an IGF-2a mRNA injected embryo (panel T). *pax2a* expression in the midbrain hindbrain boundary at the 15-somite stage in a control embryo (panel U) and an IGF-2a mRNA injected embryo (panel V). *krox20* expression in the rhombomere 3 and 5 at the 15-somite stage in a control embryo (panel W) and a 500 pg IGF-2a mRNA injected embryo (panel X). Panels A–D, lateral view, head left; panels E–H, dorsal view, head up; panels I–L and U–X, dorsal view, head left; panels M–T, front view. Black line in panels U, V indicates the gap between the midbrain hindbrain boundary and otic vesicle; black line in panels W, X indicates the gap between rhombomere 3 and 5.

We next analyzed the effect of IGF-2a ectopic expression on embryonic patterning by *in situ* hybridization using the following markers: 1) *emx1*, a telencephalon marker [20]; 2) *rx1*, a retina marker [21]; 3) *rx2*, a retina marker [21]; 4) *otx2*, an anterior neural plate marker at the bud stage and midbrain marker at the 15-somite stage [22]; 5) *pax6b*, an anterior neural plate marker at the bud stage [23]; 6) *pax2a*, a midbrain hindbrain boundary and otic vesicle marker [24]; and 7) *krox20*, a third and fifth rhombomere marker in the hindbrain [25]. Ectopic expression of IGF-2a did not alter the anterior neural plate patterning at the bud stage, as indicated by the similar expression patterns of *otx2* (Fig. 6, panels A, B, E, and F) and *pax6b* (Fig. 6, panels C, D, G, and H) between the GFP mRNA-injected control and IGF-2a mRNA injected embryos. Nor did IGF-2a expression alter the forebrain patterning, as indicated by the similar *emx1* expression patterns (Fig. 6, panels M and N). Expression of IGF-2a caused marked changes in *pax2a* expression patterns. As shown in Fig. 6, panel I, in the control embryos, *pax2a* mRNA was detected in the midbrain and hindbrain boundary (a V shaped domain) at the bud stage. In the IGF-2a mRNA-injected embryos, however, *pax2a* mRNA expressing cells remained as two separate domains at both stages (Fig. 6, panels J and V), suggesting a defect in the midline/notochord development. This effect appeared to be specific to the anterior as the *pax2a* expression in the otic vesicles was normal (Fig. 6, panel V). There were also notable changes in *krox20* mRNA expression at the bug stage. While the *krox20* mRNA expression in the third and fifth rhombomere already fused in the midline in the control embryos (Fig. 6, panel K), they remained as two separate domains in the IGF-2a mRNA-injected embryos (Fig. 6, panel L).

At the 15-somite stage, no significant difference was seen with *emx1* expression or *otx2* mRNA expressing, although the *otx2* mRNA expressing domain tended to be smaller (Fig. 6, panels M–P). Likewise, IGF-2a overexpression did not alter the expression patterns of *rx1* or *rx2* mRNA expressing pattern (Fig. 6, panels Q–T). At this stage, *pax2a* mRNA is expressed in both in the midbrain and hindbrain boundary and the otic vesicles [24 and Fig. 6, panel U]. Interestingly, at this advanced stage, *pax2a* mRNA expressing cells remained as two separate domains in the IGF-2a mRNA-injected embryos at both stages. This effect appeared to be specific to the brain region as the *pax2a* expression in the otic vesicles was normal (Fig. 6, panel V). At this stage, the *krox20* mRNA expressing cells in the third and fifth rhombomere of the hind brain already fused in the midline in the control embryos (Fig. 6, panel W), while they remained separated domains in the IGF-2a mRNA-injected embryos. Additionally, the distance between the third and fifth rhombomere was shortened in the IGF-2a mRNA-injected embryos (Fig. 6, panel X). These results suggest a possible defect in midline patterning in the IGF-2a mRNA-injected embryos.

**Figure 7:**
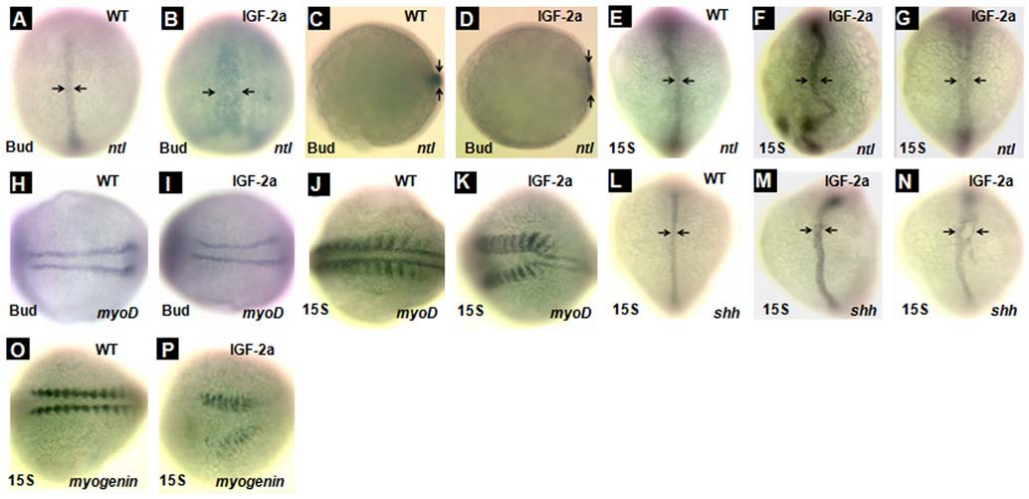
Overexpression of IGF-2a results in abnormal notochord development. *ntl* expression in the notochord at the bud stage in a control embryo (panels A and C) and an IGF-2a mRNA injected embryo (panels B and D). *ntl* expression in the notochord at the 15-somite stage in a control embryo (panel E) and IGF-2a mRNA injected embryos (panels F and G). Among the IGF-2a mRNA injected embryos, 65% showed the expression pattern shown in panel F and 35% showed that in panel G (n = 17). *myoD* expression in the somite at the bud stage in a control embryo (panel H) and an IGF-2a mRNA injected embryo (panel I). *myoD* expression in the somite at the 15-somite stage in a control embryo (panel J) and an IGF-2a mRNA injected embryo (panel K). *shh* expression in the notochord at the 15-somite stage in a control embryo (panel L) and IGF-2a mRNA injected embryos (panels M and N). Among the IGF-2a mRNA injected embryos, 71% showed expression pattern shown in panel M and 24% showed that in panel N (n = 21). *myogenin* expression in the somite at the 15-somite stage in a control embryo (panel O) and an IGF-2a mRNA injected embryo (panel P). Panels A, B, E–G, and L–N are dorsal view with head up; panels C and D are vegetal view with dorsal the the right; panels H–K, O, and P are dorsal views with head to the left.

To further investigate the possible defects in midline patterning, we performed whole mount *in situ* hybridization analysis using two midline specific markers, *shh* [26] and *ntl* [27], as well as two skeletal muscle markers, *myoD* [28]and *myogenin* [29]. As shown in Fig. 7, panels A–D, the expression domain of *ntl* mRNA at the bud stage was markedly expanded in the IGF-2a mRNA-injected embryos compared to the controls. Among the IGF-2a mRNA-injected embryos, 65% (n = 17) showed marked enlargement in the *ntl* mRNA expression domain. At the 15-somite stage, a large number of the IGF-2a mRNA-injected embryos showed abnormal *ntl* mRNA expression patterns (Fig. 7, panel F) and some even had double or partial double notochords (Fig. 7, panel G). Likewise, 71% (n = 21) of the IGF-2a mRNA-injected embryos had enlarged *shh* mRNA expression domains (Fig. 7, panel M) and 24% had double or partial double notochords (Fig. 7, panel N). In agreement with the enlarged notochord, we found that overexpression of IGF-2a increased the space between the *myoD* (Fig. 7, panels J and K) and *myogenin* (Fig. 7, panels O and P) expressing somites at the 15-somite stage. This is most evident in the anterior region of an embryo. Taken together, these results suggest that an excess of IGF signaling causes defects in the midline formation and an expansion of the notochord during early embryogenesis.

## Discussion

In this study, we have provided evidence suggesting that there are four distinct IGF genes in the zebrafish genome and all four IGF genes are expressed and functional. Although a number of partial or full-length cDNAs encoding several zebrafish IGF peptides have been documented recently, to our knowledge, our study is the first to characterize the structure of these zebrafish IGF genes, map their chromosomal loci, and identify their major transcripts. We show that zebrafish *igf-1a* has only 1 type of E-domain sequence (Ea like) but two distinct 3′UTRs. All other zebrafish *igf* genes have one 3′UTR. Both zebrafish *igf-2a* and *igf-1b* have two distinct 5′-UTR sequences, which result in two different transcription initiation sites and different signal peptides. Alternative splicing has been previously reported for mammalian IGF-1 and IGF-2 genes [30], [31]. For example human *IGF-1* has 3 distinct transcripts, which result in 3 distinct types of E-domains (Ea, Eb and Ec) [10], [32].

Among the four IGF genes, *igf-2a* and -*2b* are clearly the orthologous to human *IGF-2* and *igf-1a* is the IGF-1 ortholog. This conclusion is supported by phylogenetic analysis, detailed structural analysis, and synteny analysis results. The identity of the fourth IGF gene, however, is more enigmatic. This IGF peptide is the same to the so-called IGF-3 recently cloned from Tilapia by Wang et al. [13] recently. Zebrafish IGF-1b/IGF-3 is considerably different from the other 3 zebrafish IGF peptides. Its sequence identity with human IGF-1 is considerably lower (52%) compared to that of zebrafish IGF-1a (82%). Phylogentic analysis has placed zebrafish IGF-1b in a distinct group, independent from the IGF-1 and IGF-2 subgroups. The synteny relationship of zebrafish *igf-1b* to the human *IGF-1* is also fairly weak. We considered two possible hypothetical scenarios accounting for the origin of this particular zebrafish *igf*. One is that it represents one of the IGF-1 duplicates but it has diverged significantly from other *igf* genes after gene duplication due to its restricted expression in the gonads. The other is that it is a novel IGF as proposed by Wang et al. (2008) [13]. We favor the first scenario because it is the simplest way to interpret the data. This would mean that zebarfish *igf-1b* has diverged at a faster rate than the other IGF paralogs. Indeed, recent comparative and evolutionary biology studies using large numbers of duplicated genes in *T. rubripes*, *D. rerio*, *T. nigroviridis* and *O. latipes* have shown several cases of asymmetrical acceleration of evolutionary rate among the duplicates after the duplication [33], [34], [35]. If the second scenario were true, it would mean that one of the IGF-1 duplicates was lost after the genome duplication event and another novel IGF gene was born. Our conclusion is also in agreement with the notion that ray-finned fish have experienced a third whole-genome duplication (3R) approximately 350 million years ago. Therefore, we propose to term this gene as *igf-1b* and the encoded peptide IGF-1b.

**Figure 8:**
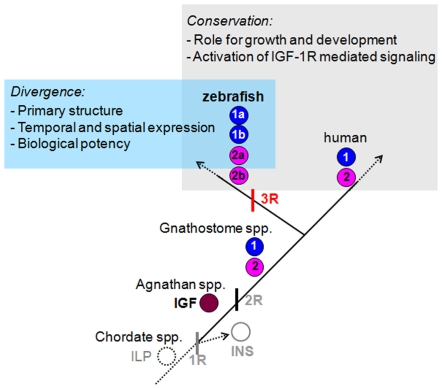
A proposed model for the evolution of multiple IGF genes in zebrafish. It is postulated that an insulin-like gene (ILP) identified in Amphioxus is the likely ancestor of modern vertebrate insulin/IGFs [2]. This ancestral gene subsequently duplicated to form distinct insulin (INS) and IGF, as found in early agnathan vertebrates [3]. The second round (2R) of whole-genome duplication gives rise to distinct IGF-1 and IGF-2 genes found in all gnathosomes [4]. The “fish specific” third round (3R) whole-genome duplication results in four distinct IGF genes, as found in zebrafish in this study. The duplicated IGF genes have evolved overlapping yet distinct functions by attaining differential regulatory mechanisms of gene expression, different peptide structure, and different biological activities.

Our finding that there are 4 *igf* genes in zebrafish and that they each encode a functional IGF ligand is interesting when considering the evolution of the insulin/IGF gene family. There are a number of published studies on the evolution of this important family. Chan et al. (1990) has characterized an insulin-like molecule from the protochordate amphioxus [2]. This molecule contains features characteristic of both vertebrate insulin and IGF, and therefore may represent a modern version of the ancestral molecule for the insulin/IGF gene family [2]. In the Atlantic hagfish, an agnathns, 2 members of the IGF/insulin gene family have been identified - one is an insulin ortholog and the other is IGF-like. The hagfish IGF is equally related to human IGF-1 and IGF-2, and may therefore be a prototypical IGF molecule [3]. Duguay et al. (1995) have shown that the spiny dogfish, an elasmobranch species, has an IGF-1-like molecule and an IGF-2-like molecule, suggesting the prototypical IGF molecule diverged in an ancestor of the extant ganathostomes [4]. A large number of studies have shown that two distinct IGF genes (IGF-1 and IGF-2) and an insulin gene are present in most vertebrates, including birds, and mammals [1]. Based on the previous studies by others and the current study, we speculate that a single ancestral insulin/IGF molecule is present in protochordate animals. The first genome duplication (1R) that presumably occurred 500 million years ago in metazoans, might have resulted in the appearance of a distinct insulin molecule and an IGF molecule in agnathans. The second round whole-genome duplication (2R) that occurred 400 million years ago, might have given the rise to separate IGF-1 and IGF-2 genes. The “fish specific” or third round whole-genome duplication (3R) that occurred approximately 350 million years ago might have resulted in two distinct *igf* genes in zebrafish and probably other teleost fishes (Fig. 8). The retention of these IGF genes in the zebrafish genome suggests that each of them may have evolved unique and indispensable roles. This notion is supported by a recent study reporting distinct embryonic roles of zebrafish IGF-2a and -2b [12]. In this study, we provide further evidence that these zebrafish IGF genes have evolved overlapping yet distinct biological properties by attaining distinct temporal expression patterns, different peptide structure, and different biological activities (Fig. 8). The duplicated *igf-1* and duplicated *igf-2* appear to have diversified in several ways. First, each of these IGF ligand genes exhibits distinct spatial and temporal expression patterns. During early development, IGF-1b and IGF-2a mRNAs are detected throughout the embryogenesis, while IGF-1a and IGF-2b mRNAs are detectable only after the gastrula stage. In the adult, IGF-2a mRNA is found in all tissues examined with the exception of the muscle and gill, while IGF-2b mRNA is only expressed in the liver. In comparison, IGF-1a mRNA is detected in the liver, brain, and eyes. IGF-1b mRNA, on the other hand, is exclusively expressed in the ovary and testes. Furthermore, these 4 IGF peptides have clear differences in their primary structure and biological activities. When tested in zebrafish embryos by mRNA injection experiment, all resulted in the activation of Akt signaling and had biological effects in developing zebrafish embryos. These findings make an important advance in our understanding of the structural and functional evolution of the IGF/insulin family.

We have previously reported that zebrafish contain two functional *igf-1r* genes [5], [16], [36], two functional *igfbp-1*, two *igfbp-2*, and two *igfbp-5* genes in zebrafish [37], [38]. In this study, we have shown that there are two functional *igf-1* and two *igf-2* genes in zebrafish. Therefore, it appears that most, if not all, components in the IGF signaling pathway are retained as duplicates in the zebrafish genome. The retention of these IGF genes indicates that each of them may have evolved unique and indispensable roles. One intriguing finding made in this study is that after duplication each pair has evolved very distinct expression profiles. While zebrafish IGF-1a mRNA is easily detected in multiple adult tissues in zebrafish, IGF-1b mRNA is only found in the gonad. Likewise, IGF-2b mRNA is only detected in the liver whereas IGF-2a mRNA is expressed in most adult tissues. During embryogenesis, IGF-1b and IGF-2a mRNAs are detected from the 1-cell stage and throughout embryogenesis, whereas IGF-1a and IGF-2b mRNAs become detectable after the gastrula stage. In mammals, IGF-1 mRNA is highly expressed in the adult liver under the control of growth hormone (GH). IGF-1 mRNA is also expressed in many non-hepatic tissues and that extra-hepatic sources of IGF-1 can compensate for the absence of hepatic IGF-1 production in mice [39]. In juvenile and young salmon and trout, detectable amounts of IGF-1 mRNA are found in virtually all tissues examined, but the liver has the highest levels, suggesting that the liver is the major site of IGF-I mRNA production, as is the case in mammals and birds [40], [41], [42]. There is good evidence that GH regulates IGF-1 expression in teleost fish [43], [44], [45], [46]. In mammals, IGF-2 is an important fetal growth factor and the IGF-2 gene is highly expressed in the mammalian fetus [47]. In rodents, the IGF-2 gene is highly expressed in the fetus [47]. Similarly, abundant IGF-2 mRNA is detected in fish embryos and larva [48]. Considerable interspecies variations are found during postnatal or posthatching regulation of IGF-2 gene expression. In the rat, the IGF-II gene expression declines after birth in all tissues except for choroid plexus and leptomenings, whereas in humans and guinea pigs levels of IGF-II mRNA remain high in many adult tissues [47]. Among the 4 zebrafish IGF genes, IGF-1a and IGF-2a are expressed in multiple adult tissues in zebrafish, resembling the profiles of their mammalian orthologs. In contrast, zebrafish IGF-1b mRNA is detected only in the gonad and IGF-2b mRNA in the liver. These data suggest that the duplicated IGF genes have undergone subfunction partitioning in their expression.

The finding that *igf-2b* is exclusively expressed in the liver of adult zebrafish is intriguing in light of the previous reports that IGF-2 mRNA levels are high in the adult liver and other tissues in rainbow trout and seabream [42], [48] and that GH treatment significantly increases the IGF-2 mRNA levels in the liver in rainbow trout [46]. This raises the interesting possibility that IGF-2a may be a major form of IGF ligand in the circulation in adult zebrafish. The gonad-specific expression of *igf-1b* in zebrafish is in good agreement with a recent report in tilapia [13]. IGF-1 has been shown to act in a paracrine fashion to regulate ovarian steroidogenesis in humans [49]. IGF-1 plays an important role in mammalian testicular function. Fish ovaries express IGF-1 mRNA [44], [50] and have specific binding sites for IGF-1[51]. IGF-I binding to fish ovaries activates receptor tyrosine kinase activity and increases gonodotropin II-stimulated steroidogenesis in salmon granulosa cells [51]. A role of IGF-1 in inducing final oocyte maturation has also been demonstrated in fish [52]. The discovery of a gonad-specific IGF gene in teleost fish highlights the importance of local IGFs in the gonad and opens new avenues for future studies.

IGF signaling is widely considered a key growth regulator in fetal and postnatal stages [1], [53]. This dogma is primarily derived from mouse genetic studies. Overexpression of IGF-1 in mice increases the body weight by 30% [54]. IGF-I or IGF-II knockout mice had birth weights about 60% that of their littermates, while IGF-I/IGF-II double knockout mice were even smaller, weighing only 30% of their littermates [55], [56]. The IGF-IR knockout mice had a birth weight 45% of their littermates [55], [56]. The importance of IGFs in growth is also supported by the growth-retarded phenotype resulting from mutations in human IGF-I or IGF-IR genes. A homozygous partial deletion in the human IGF-I gene is associated with fetal and postnatal growth retardation [57]. Patients with point mutations in the IGF-IR gene exhibited severe intrauterine growth restriction and poor postnatal growth [58]. Recent studies in lower vertebrates suggested that IGF signaling may play additional roles during early development. In Xenopus, ectopic expression of IGF-2 was shown to induce anterior neural tissues [59]. Inhibition of IGF signaling using a dominant-negative IGF-IR or antisense morpholino reduced anterior tissues in *Xenopus* [59], [60]. Contradictory evidence has been reported on this issue in zebrafish. Eivers et al. (2004) reported that overexpression of a secreted form of IGF1R (a truncated IGF-1R lacking both the transmembrane and intracellular domains) in zebrafish resulted in defects in anterior neural structure and overexpression of IGF-1 resulted in expansion in anterior structures at the expense of trunk and tail [61]. Loss-of-function studies in zebrafish, however, showed that suppression of IGF signaling by a dominant-negative IGF-IR or antisense morpholinos did not result in any anterior/neural patterning defects [16], [36]. Likewise, knockdown of zebrafish *igf-2a* and/or *igf-2b* did not cause defects in anterior neural patterning [12]. In this study, we have shown that an excess of IGF signaling did not cause notable anterior defects in zebrafish. These mRNAs encoding various IGF ligands were clearly functional because they induced Akt phosphorylation and caused morphological changes in developing zebrafish embryos. The lack of notable anterior/neural patterning defects in zebrafish revealed by previous loss-of-function studies [12], [16], [36] and the current gain-of-function study strongly argue against a critical role for IGF signaling in anterior/neural induction in zebrafish.

A major finding made in this study is that IGF signaling plays a critical role in midline and notochord development. When tested in zebrafish embryos by mRNA injection experiments, all 4 zebrafish IGFs resulted in delayed mesoderm involution, poorly formed notochords and widened distance between somites. Our results indicate that ectopic expression of zebrafish IGF-2a or any IGF ligands, resulted in defects in the midline formation and an expansion of the notochord. These morphological changes are associated with a marked expansion in the *ntl* mRNA expression domain and *shh* mRNA expression domain. Overexpression of IGF-2a also caused a widening in the space between the *myoD* and *myogenin* in the anterior region of an embryo. Many of these embryos had duplicated or partial duplicated notochords, suggesting that an excess of IGF signaling causes defects in the midline formation and an expansion of the notochord. Brown et al. (2009) reported that knockdown of either *igf-2a* of *igf-2b* led to defects in dorsal midline development, although knockdown of *igf-2b* also induced ectopic fusion of the nephron primordia [12]. Collectively, these studies suggest that that IGF signaling plays a critical role in midline formation and notochord development. These results not only provide new insights into the functional conservation and divergence of the multiple *igf* genes but also reveal a novel role of IGF signaling in midline formation and notochord development in a vertebrate model. Given the central role of IGF signaling in growth regulation, it will be important to investigate whether this high rate of gene retention in genes belonging to the IGF signaling pathway in the ray-fin fish plays any role in the continuous growth pattern of the teleosts.

## Materials and Methods

### Chemicals and reagents

All chemicals and reagents were purchased from Fisher Scientific (Pittsburgh, PA) unless otherwise noted. Restriction endonucleases were purchased from New England BioLabs (Beverly, MA). Superscript II reverse transcriptase (RT) and Oligonucleotide primers were purchased from Invitrogen Life Technologies, Inc. (Carlsbad, CA). RNase-free DNase was purchased from Promega (Madison, WI). The anti-Akt and anti-Phospho-Akt antibodies (Ser473) were purchased from Cell Signaling (Danvers, MA).

### Experimental animals

Zebrafish (*Danio rerio*) were maintained at 28 C on a 14 h∶10 h (light∶dark) cycle, and fed twice daily. Embryos were generated by natural crosses. Fertilized eggs were raised in embryo medium at 28.5 C and staged according to the standard criteria of Kimmel et al. [62]. For in situ hybridization analysis, embryo medium was supplemented with 0.003% (w/v) 2-phenylthiourea to inhibit embryo pigment formation. All experiments were conducted following guidelines approved by the University of Michigan Committee on the Use and Care of Animals.

### Molecular cloning

Searching the zebrafish EST and genome databases (Ensembl Zebrafish: http://www. ensembl.org/Danio_rerio/blastview) and the NCBI database (http://www.ncbi.nlm.nih.gov/) identified two distinct IGF-2 mRNAs (GenBank Accession No. XM_001338006, 3404 bp; NM_131433, 2065 bp, respectively) and one IGF-1 mRNA (GenBank Accession No. NM_131825, 1521 bp). We further searched these databases by BLAST searches using the IGF-1 sequence as a query. Two IGF-1-like EST sequences (GenBank Accession No. XM_684982, 636 bp; CO353655, 600 bp, respectively) were obtained. Based on these sequences, the following gene-specific primers were designed to clone and confirm the predicted IGF cDNA sequences.

***igf-1a*** **:** 5′ RACE primer 5′- TGTCTGCATAAGGCTTTTGAATAATCC-3′, 5′ nest RACE primer 5′-CTGTCGGTTTGCTGAAAT-3′; 3′ RACE primer: 5′- ATAGCCACTCTTCCTGTAAGG-3′ and 3′ nest RACE primer 5′- TAGAGGACAGCGGGAGGAATGAACTGA-3′.

***igf- 1b*** **:** 5′ RACE primer 5′-AAGCCYCTGTCTCCRCACACAAACTSCAG-3′, 5′ nest RACE primer 5′- TCTGGCCCCTTCAGTGTTGTCTGGCAG-3′; 3′ RACE primer: 5′- CTGSAGTTTGTGTGYGGAGACAGRGGCTT-3′ and 3′ nest RACE primer 5′- CGACAACTGAGACGCCAACA-3′.

***igf-2a*** **:** 5′ RACE primer 5′- CATACATCGCGTGTCTTACATACACGT-3′, 5′ nest RACE primer 5′- CTCCKTARCCTCTGAGCAGC -3′; 3′ RACE primer: 5′- CCATCCTTCTTCCCACAG-3′ and 3′ nest RACE primer 5′- TGAAAAGACAGGATCGTTTGCACAGAG-3′.

***igf-2b*** **:** 5′ RACE primer 5′- AGCAAAGGCTTTAACTTCATTCTCCA-3′, 5′ nest RACE primer 5′- CTCCKTARCCTCTGAGCAGC -3′; 3′ RACE primer: 5′- CAACCTGCCATCGTCCCA-3′ and 3′ nest RACE primer 5′- TGATGGCCAGCCATCCCAAATGGCA-3′. Total RNA was isolated from 10 hpf zebrafish embryos and adult liver using the TRIzol reagent (Invitrogen) and used as template. The 5′ and 3′ ends of the IGF mRNA were amplified using a SMART RACE cDNA amplification kit (Clontech, USA) following the manufacturer's protocol. PCR products were gel purified and ligated into the T/A cloning vector pGEM-T Easy (Promega, USA) and transformed into *E. coli* DH5a competent cells. Positive clones were examined by PCR and direct sequencing.

### Phylogenetic analysis and physical mapping

The genomic structure and chromosomal location of each zebrafish *igf* gene were determined by searching the zebrafish genome (http://www.ensembl.org/Danio_rerio/index.html). Amino acid sequences of IGFs were aligned by CLUSTAL X. Phylogenetic analysis was done using B and A domain sequences by Neighbor-joining method in the MEGA3 program. Gap sites in the alignment were not used in the phylogenetic reconstruction. The reliability of the estimated tree was evaluated by the bootstrap method with 1000 pseudo-replications. Synteny analysis was carried out based on Danio rerio Zv6 and Homo sapiens Build 36.3 (http://www.ensembl.org/Homo_sapiens/index.html) and data from zebrafish and human synteny map. Genomic structure was determined by searching the zebrafish genome (http://www.ensembl.org/Danio_rerio/index.html).

### Plasmid construction

The open reading frame of each zebrafish IGF gene was amplified by PCR using *Pfu* DNA polymerase (Stratagene, La jolla, CA, USA). The following primers were used: *igf-1a* 5′- GTGGATCCACCATGTCTAGCGGTCATTTCTTCCAG-3′ and 5′-GCTCTAGATCACATGCGATAGTTTCTGCCCCCTGTGT-3′, *igf-1b* 5′- GTGGATCCACCATGGTGCCCAGCTGGCGGAGTGT-3′ and 5′- GCTCTAGATCACCGGATGTGAGAGATGAATGTTGGCGT-3′, *igf-2a* 5′- GTGGATCCACCATGGAGGACCAACTAAAACATCAT-3′and 5′- GCTCTAGATCACTTGTGGCTAACGTAGTTTTCTGTGG-3′, *igf-2b* 5′- GTGGATCCACCATGGATGATTACCATGTATTCTGTGC-3′and 5′- GCTCTAGATCATTTTCGGGATGTGCTGATCTGGACGT-3′. The resulting cDNA was cloned into the pGEM-T Easy vector (Promega, USA). To generate the IGF peptide expression constucts, the above PCR product was digested with *BamHI* and *XbaI* and subcloned into *BamHI* and *XbaI* sites of pCS2+.

### Reverse transcription (RT)-PCR and whole mount *in situ* hybridization

Total RNA was isolated from embryo or adult tissues using the TRIzol reagent (Invitrogen). One µg of RNA was reverse-transcribed to single strand cDNA using SuperScript II reverse transcriptase according to the protocol. The following primers were used for the PCR amplifications: *igf-1a*, 5′- AGCGGTCATTTCTTCCAG-3′ and 5′- CCTTACAGGAAGAGTGGCTAT -3′; *igf-1b*, 5′- GTGCTGCGTTCTCATCCT -3′ and 5′- TGTTGAGGAGGTTTGGGT -3′; *igf-2a*, 5′- TAACCCTGTCTGCCTTCG -3′ and 5′- CTGTGGGAAGAAGGATGG -3′; *igf-*2b, 5′- TCAAACAGCCGCCGTCCTC -3′ and 5′- TGGGACGATGGCAGGTTG -3′; *β-actin*, 5′- CTTGCGGTATCCACGAGAC -3′ and 5′- GCGCCATACAGAGCAGAA -3′. PCR cycles and amounts of templates were optimized for each primer set in pilot experiments. PCR cycles were as follows: 95°C for 3 min, 34 cycles of 94°C for 30 s, 53°C (*igf-1a* and *igf-1b*) or 56°C (*igf-2a*, *igf-2b*, and *β-actin*) for 30 s, and 72°C for 1 min. PCR products were analyzed by electrophoresis followed by ethidium bromide staining and a no-template control was the negative control.

Whole mount *in situ* hybridization using digoxigenin (DIG)-labeled RNA riboprobe was carried out essentially as reported previously [63]. Each template plasmid was linearized by restriction enzyme digestion, followed by *in vitro* transcription reactions with T7 or Sp6 RNA polymerase to generate the antisense or sense RNA riboprobes. Embryos were hybridized with the appropriate riboprobe at 65°C, followed by incubation with an anti-DIG antibody conjugated with alkaline phosphatase and stained with substrate NBT/BCIP to produce purple insoluble precipitates. Photographs were taken with a Nikon EC600 fluorescence microscope (Melville, NY).

### Microinjection experiments

Capped mRNA was synthesized using a commercial kit and linearized plasmid DNA as template (mMESSAGE mMACHINE kit; Ambion, Inc.). mRNA (500 pg per embryo) was microinjected into zebrafish embryos at the 1-2 cell stage as reported previously [36]. GFP mRNA injected and wild type embryos were used as controls. After injection, embryos were placed in embryo rearing medium and kept at 28.5°C.

### Western immunoblot

Twenty embryos from each treatment group were dechorionated, deyolked, and homogenized in 40 µl of RIPA buffer (50 mM Tris-HCl, 150 mM NaCl, 2 mM EGTA, 0.1% Triton X-100, pH 7.5) containing 10 µg/ml aprotinin, 10 µg/ml leupeptin, 10 µg/ml pepstatin, and 100 mM PMSF. The homogenates were briefly centrifuged to pellet cellular debris and the supernatant was retained. Protein levels of each sample were quantified using a protein assay kit (Pierce Biotechnology, Rockford, IL). Equal amounts of protein were analyzed by SDS-PAGE and Western immunoblot as previously described [36]. Antibodies against total Akt or the phospho-Akt (Ser473) were used at a 1∶1000 dilution.

### Statistics

Values are means±S.E. Differences among groups were analyzed by one way ANOVA followed by Fisher's Post Hoc tests or unpaired t-test. Significance was accepted at p<0.01.

## Acknowledgments

We thank Mr. John Allard for proof reading this manuscript.
